# A Nonthoracotomy Myocardial Infarction Model in an Ovine Using Autologous Platelets

**DOI:** 10.1155/2013/938047

**Published:** 2013-12-03

**Authors:** Tyler Spata, Daniel Bobek, Bryan A. Whitson, Sampath Parthasarathy, Peter J. Mohler, Robert S. D. Higgins, Ahmet Kilic

**Affiliations:** ^1^The Ohio State University Wexner Medical Center, Columbus, OH 43210, USA; ^2^Division of Cardiac Surgery, Department of Surgery, The Ohio State University Wexner Medical Center, 410 W. 10th Avenue, N-831 Doan Hall, Columbus, OH 43210, USA

## Abstract

*Objective*. There is a paucity of a biological large animal model of myocardial infarction (MI). We hypothesized that, using autologous-aggregated platelets, we could create an ovine model that was reproducible and more closely mimicked the pathophysiology of MI. *Methods*. Mepacrine stained autologous platelets from male sheep (*n* = 7) were used to create a myocardial infarction via catheter injection into the mid-left anterior descending (LAD) coronary artery. Serial daily serum troponin measurements were taken and tissue harvested on post-embolization day three. Immunofluorescence microscopy was used to detect the mepacrine-stained platelet-induced thrombus, and histology performed to identify three distinct myocardial (infarct, peri-ischemic “border zone,” and remote) zones. *Results*. Serial serum troponin levels (**μ**g/mL) measured 0.0 ± 0.0 at baseline and peaked at 297.4 ± 58.0 on post-embolization day 1, followed by 153.0 ± 38.8 on day 2 and 76.7 ± 19.8 on day 3. Staining confirmed distinct myocardial regions of inflammation and fibrosis as well as mepacrine-stained platelets as the cause of intravascular thrombosis. *Conclusion*. We report a reproducible, unique model of a biological myocardial infarction in a large animal model. This technique can be used to study acute, regional myocardial changes following a thrombotic injury.

## 1. Introduction

Myocardial infarction (MI) resulting from coronary arterial disease is the number one cause for mortality in the United States. It is estimated that roughly 785,000 people in the United States will have a new MI in 2012 with an additional 470,000 having a recurrent MI [1, 2]. Early diagnosis and treatment of myocardial infarction is crucial with optimal outcomes in patients seeking immediate medical attention [3].

Currently, there are several large animal models used to study myocardial infarction; however, they deviate significantly from the biological pathophysiology of human MI [4–6]. In our present experiments, we illustrate the use of autologous-aggregated platelets to create an ovine model of MI that is reproducible and more akin to the natural MI process in humans.

## 2. Materials and Methods

All studies were conducted with approval by the Institutional Animal Care and Use Committee (IACUC) at Ohio State University (Study number: 2012A00000040). Adult male Dorsett sheep weighing between 50–70 kg were used for this study (*n* = 7). Strict adherence was kept to the Guide for the Care and Use of Laboratory Animals of the National Institutes of Health.

### 2.1. Mepacrine Labeled Platelet Aggregates

Autologous platelet aggregates were made by collecting 200 mL of venous blood 24 hours prior to the embolization procedure. The blood was drawn in a sterile fashion, centrifuged at 200 ×g for the supernatant, then 1500 ×g for platelet isolation. Platelets were stabilized with a buffer (pH 6.5) consisting of 6.85 mM citric acid, trisodium salt in saline [4]. Mepacrine (10 mM) in phosphate buffered saline (PBS) was used to stain the platelets for 1 hour prior to the embolization experiment and to confirm presence of platelet induced thrombus in infracted tissue. The platelets were then stored in 15 mM Trizma and saline buffer (pH 7.4) as a thrombus was then created by adding 25 *μ*L of thrombin to the platelets in a 3 mL syringe. The syringe was then wrapped in aluminum foil to limit further enzymatic reaction and stored at ^+^4°C for creation of the thrombus for injection the following day. The mepacrine-stained platelets were verified in the tissue under a Zeiss LSM 510 Confocal Microscope with Argon-2 laser at 488-nm excitation with 500–530-nm BP filter at 63x.

### 2.2. Serum Troponin Levels

Serum was collected daily at baseline and at postprocedure day 1, 2, and 3 for troponin level assessment [7]. A volume of 0.25 mL of plasma was processed in the Ohio State University Chemistry Lab (Columbus, OH) for troponin levels (*μ*g/mL) at each time point (ADVIA Centaur XP Immunoassay System, Global Siemens Healthcare Sector, Germany).

### 2.3. Embolization Procedure

All animals were sedated, and all attempts were made to minimize animal discomfort as previously described [4]. A right lateral decubitus position was utilized with the left neck being clipped, prepped, and draped in standard sterile, surgical fashion.

To minimize potentially fatal arrhythmias and provide hemodynamic support during the peri-infarct period, intravenous inotropic (epinephrine at 0.25 mcg/kg/hr) and antiarrhythmic (lidocaine at 2 mg/hr) medications were used [8].

The left carotid artery was surgically exposed, and an introducer sheath (8F Axcess, Argon Medical Devices, Athens, TX) was placed. Subsequently, under fluoroscopic guidance (GE-OEC 9800 Plus, Salt Lake City, UT) a Wiseguide 8 French AL-1 guide catheter (Boston Scientific, Maple Grove, MN) was placed into the left main coronary artery. Five-20 cc of Omnipaque (iohexol) Injection Contrast (350 mgI/mL, GE Healthcare Inc., Princeton, NJ) was used for preembolization verification of the coronary anatomy, and all images were digitally stored. A Bard, Tru-Trac 4 mm × 2 cm balloon catheter (Covington, GA) was positioned in the mid-left anterior descending (LAD) coronary artery. A balloon catheter was inflated to 2–4 atmospheres using an Encore 26 inflation device (Boston Scientific, Cork, Ireland) to temporarily occlude the LAD and to prevent reflux during the infusion. Aggregated platelets (1.5 mL) were infused through the balloon catheter guide wire lumen to infarct the LAD. Subsequently, 5 mL of nonheparanized saline was used to flush the catheter, and then the balloon was deflated after 30 seconds.

Continuous electrocardiograms and arterial line hemodynamic monitoring was carried out with a postembolization angiogram verifying occlusion of the mid-LAD. The epinephrine and lidocaine drips were weaned over a period of twenty minutes. The incision was then closed, and the animal was allowed to recover.

### 2.4. Procurement Procedure

On post-embolization day 3, the sheep underwent a 5th intercostal space left thoracotomy, and a cardiectomy was performed [4]. Standard double-staining technique was utilized for infarct size measurement with imaging software readily available (ImageJ 1.46r, Wayne Rasband, National Institutes of Health, Bethesda, MD) [5]. Different regions of the left ventricle that were harvested including the (1) infarct zone, (2) peri-ischemic border zone, and (3) remote zones were identified. The infarct zone was identified by location of the mepacrine-staining thrombus injection on the animal's respective angiogram as well as the grossly stained myocardium after the tissue harvest (i.e., significant fibrosis present, pale myocardial surface, and thinning of the myocardium wall). The peri-ischemic border zone was identified as the area between 0.5 and 2.0 cm away from the edge of grossly stained myocardium. The remote zone was identified by being at least 3 cm away from the edge of grossly infracted tissue. All regions were verified by histological and staining techniques.

### 2.5. Statistical Analysis

All measurements are expressed as mean ± standard error of the mean. A repeated measures analysis of variance (ANOVA) was used to compare the serial daily intragroup troponin-level trends following embolization. A retrospective power analysis was performed to evaluate the power of the study and verify the sample size. Calculations were performed by using IBM SPSS Statistics for Windows, Version 19.0 (IBM Corp., Released 2010, Armonk, NY).

## 3. Results

### 3.1. Reproducibility of Infarction

**Figure 1:**
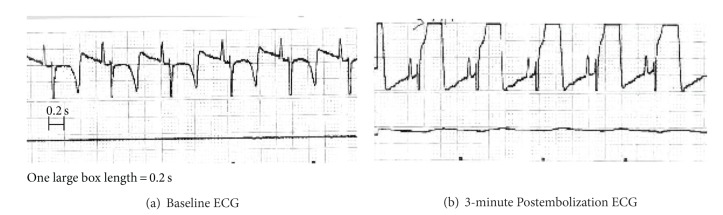
Electrocardiogram (ECG) readings during embolization. Myocardial infarction verification through electrocardiogram changes. Representative electrocardiogram (ECG) readings from Lead II during the embolization procedure in one animal showing (a) baseline, or preembolization ECG showing absence of ischemia, and (b) 3 minute-post embolization showing “tombstone” or significantly elevated ST segments consistent with a large anterior infarction.

Five of seven animals survived resulting in a twenty-eight percent attrition rate secondary to lethal arrhythmias and/or cardiogenic shock. The other five animals survived to completion of the study. All animals had significant ST elevation in the continuous single lead electrocardiogram monitoring during the procedure as illustrated in Figure 1. Additionally, a single distinct episode of myocardial injury was verified with serial daily serum troponin levels showing a peak level at postembolization day 1 of 297.4 ± 58.0 *μ*g/mL (see Table 1).

**Figure 2:**
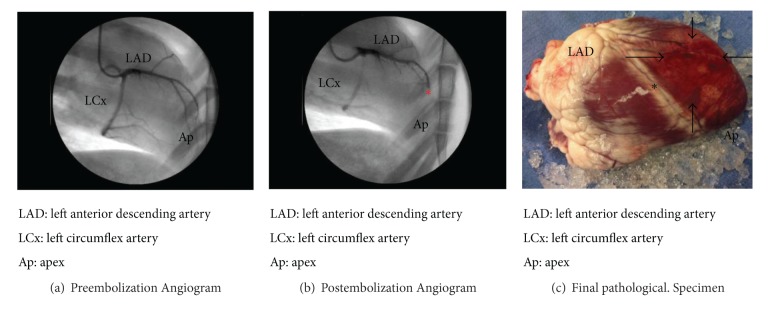
Pre- and postembolization angiograms and infarct area at cardiectomy. Illustrative example of embolization and corresponding area of infarctionat cardiectomy. (a) Preembolization angiogram showing the relationship of the nativeovine coronary anatomy and selection of embolization target of the LAD. (b) Area of thrombus formation immediately postembolization (note that ∗ corresponds to the location of embolization injection). (c) Final pathology showing the corresponding area of infarction (note that ∗ once again shows area of embolization with arrows demarcating the area effected by infarction).

A representative angiogram of pre- and post-embolization with corresponding infarct size at post-embolization day 3 is shown in Figure 2. The catheter directed embolization was carried out at the mid to distal one-third of the LAD, and double staining technique revealed an infarct size of 35.8 ± 3.5% of the left ventricular free wall.

### 3.2. Histology

**Figure 3:**
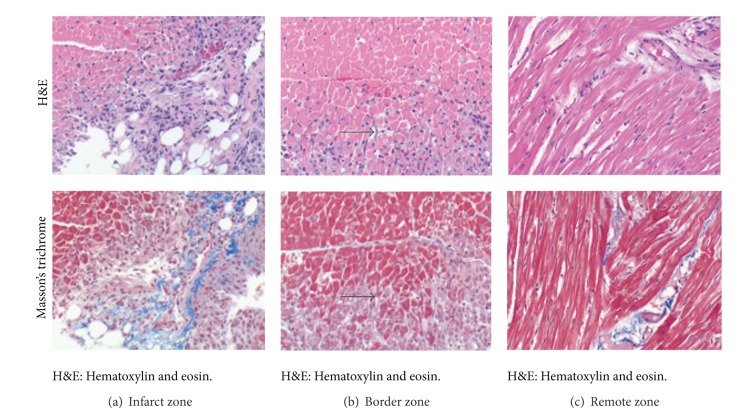
Histological analysis highlighting the three distinct zones of myocardium post-embolization. Representative regional myocardial histology (20x magnification). (a) Infarct tissue showing significant area of inflammation (H&E) and fibrosis (Masson's trichrome), (b) border zone myocardium showing the transition (delineated by blocked arrows) between normal myocytes (top) and ischemic tissue (bottom), and (c) remote zone showing normal myocardial cell structure and no fibrosis.

**Figure 4:**
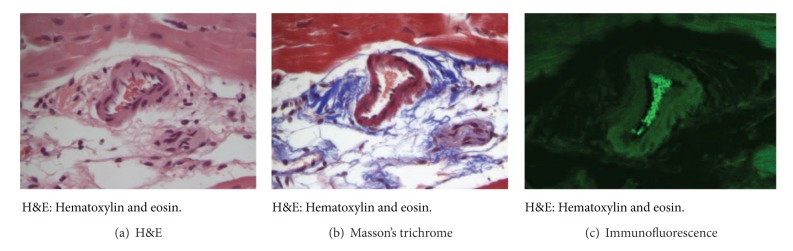
Intravascular thrombus confirmation with mepacrine-labeled platelets. Representative intravascular thrombus secondary to mepacrine-labeled platelet embolization (magnification 63x). (a) H&E showing inflammation and intravascular thrombus. (b) Masson's trichrome showing early fibrosis and organization of immature collagen deposition. (c) Immunohistochemistry showing intravascular presence of mepacrine labeled platelets.

Hematoxylin and eosin staining as well as Masson's trichrome staining confirmed three distinct regions of myocardial cell hypertrophy, inflammation, and apoptosis. The regions of (a) noninfarcted zone (>3 cm from the region of infarct), (b) peri-ischemic border zone (defined as within 0.5–2.0 cm of the gross edge of ischemic tissue), and (c) the infarct zone are illustrated in Figure 3. The remote zone myocardium shows little to no inflammatory cells present. Within the peri-ischemic border zone, there is an area of demarcation between the presence of inflammatory cells and tissue spared of inflammation and ischemia. The infarcted area has little viable cardiomyocytes present on postembolization day 3. Immunofluorescence verifies the presence of intravascular mepacrine-stained platelets as the cause of thrombosis as seen in Figure 4.

## 4. Discussion

### 4.1. Current Models and Their Limitations

Large animal models make it more challenging to provide a direct ischemic event in the myocardium without either a thoracotomy with direct ligation or embolization using synthetic, nonbiological beads [6]. Indeed, the currently available models of heart failure are initiated by either (1) rapid-ventricular pacing induction, (2) aortic banding, (3) intracoronary injection of ethyl alcoho1 [9], (4) intracoronary balloon occlusion [10], or (5) surgical occlusion of coronary arteries [11]. Other ways to induce catheter-based ischemia include ameroid constrictors and coiling/gelfoam in addition to cryonecrosis within the coronary artery after a thoracotomy [6]. All of these methods deviate significantly from the normal mechanisms of human MI [4].

Although the direct coronary artery ligation model is widely used in large animals, MI in humans is due to a thromboembolic event and not a direction ligation of a coronary artery with an accompanying surgical trauma. Other less invasive animal models (i.e., injection of polystyrene beads into the coronary artery causing myocardial injury to the left-ventricle) have more closely mimicked the human pathophysiologic process; however, the use of foreign materials, such as the polystyrene beads, can potentially stimulate an exaggerated inflammatory response making it a less than ideal model for study [4]. Recent research has shown that platelets in the thromboembolic events do trigger initial inflammatory reactions with microembolization after myocardial injury [12]. With our alternative, nonsurgical model of permanent coronary artery occlusion, we can more closely recreate the thromboembolic phenomenon that occurs during the human MI. In addition, the ability to localize and reproduce the area of injury gives us the ability to study not only the global cardiac changes but also the local changes that occur after an MI.

### 4.2. Current Study on the Ovine Model

Our current, nonthoracotomy strategy of accomplishing myocardial infarction should enable us to more closely explore the cellular and molecular pathways underlying an acute MI. It is essential to improve our current preclinical models to gain a better understanding of the human pathophysiology. This approach is important in order to accurately develop a relevant pathophysiologic model for clinical therapeutic evaluations. Unfortunately, there currently is a paucity of a biologic large animal model of an acute MI. Mice models of myocardial ischemia and heart failure are most commonly employed due to cost efficiency and availability; however, these models do not necessarily translate into the human condition.

Various different species of large animals (i.e., dogs, pigs, and sheep) have been used to explore myocardial disease. Amongst the large animal models, the ovine model has been shown to more closely mimic human coronary arterial anatomy [6, 13]. The sheep are superior to canines in the manner with which the heart behaves towards coronary arterial injury [14] and are less arrhythmogenic than pigs to myocardial infarction [15].

Sheep, however, do have some significant variability in the distribution of their left circumflex artery, leading to significant variation in infarct sizes [16]. Our group had previously reported on the efficacy of repeated embolizations to the left circumflex artery to induce heart failure [4]. Subsequently, we have noted that the left anterior descending (LAD) artery tends to be less variable and hence have elected to modify our techniques to create a more reproducible model of an acute myocardial infarction using the LAD as the target for biological embolization. Previous ovine models of postinfraction heart failure through the ligation of either the distal LAD or the diagonal branches off of the LAD have shown reproducible results. This has been attributed to the consistent territory of myocardium supplied by this artery as well as the lack of collateral vascularity [16–18].

Various studies have been performed to evaluate the importance of how different regions of the heart are affected by a myocardial infarction. After a localized infarction, there is an increase in regional remodeling strain as a result of increased myocardial apoptosis and regional contractile dysfunction [19]. Multiple regional coronary ligations have been shown as a model to mimic global ischemic cardiomyopathy [20].

Clinically, various studies have shown the importance of not only regional but also global cardiac changes that occur after a localized myocardial infarction, governed by local changes in blood flow as well as global changes in adjacent myocardium [21]. The heart must undergo a variety of complex pathways, especially in ways to heal from the infarction [22–24]. Such cardiac remodeling pathways as calcium-handling [25–28], inflammation [28–31], angiogenesis [28, 31], hypertrophy [26, 30, 32], and cell survival/apoptosis [25, 26, 29–32] are still being studied in how the myocardial compensates to episodes of ischemia with the eventual development of heart failure. The only way to better study these regional changes is to develop a large animal model that closely mimics the human myocardial infarction pathophysiology.

### 4.3. Limitations

There are limitations to this study. The ovine left coronary anatomy does have some variation; the different anatomical configurations include (1) origin of where left circumflex branches result in a short left main coronary artery, (2) complex angles amongst the left main coronary artery requiring longer procedure duration, and (3) high bifurcation to an LAD diagonal branch that provides significant blood flow to the left anteroseptal area of the heart (and thus making the heart more prone to malignant arrhythmias). Our goal was to place the embolization catheter/balloon past the last prominent diagonal branch of the LAD to prevent occlusion of the branch and better localize the area of ischemia. However, the LAD diagonal variability results in a variation of the amount of myocardial ischemia resulted from the embolization. The previous model [4] used the left circumflex artery in order to provide adequate myocardial tissue on the anterior portion of the heart for future studies with left-ventricular assist devices. In addition, although the thrombus size appeared consistent and was measured in a consistent aliquot, the thrombus had an organic appearance making it challenging to quantify absolutely.

### 4.4. Future Implications

The ideal embolization model should address variations of LAD anatomy to provide consistent areas of occlusion following embolization. Thus, future studies should analyze regional versus global mRNA/protein expression amongst the different myocardial zones following an infraction. Possible therapeutic interventions, including regionally targeted anti-inflammatory, proangiogenic, and antiapoptotic measures, can be tested using this model without the added variability of surgical and/or nonbiological inflammatory insult. As we further test the hypotheses with this model, we move the field closer to an ideal model to study acute human myocardial infarction.

## 5. Conclusion

Our unique, nonthoracotomy surgical model platform will enable investigators to identify regional as well as global changes that occur after an acute heart attack. These targeted myocardial zones will enable the identification of genetic and protein targets to prevent infarct expansion following an MI. It is our hope that this approach will help to mitigate maladaptive cardiac remodeling and stop the development of ischemic heart failure in future studies.

## Figures and Tables

**Table 1:** Serial serum troponin levels.

	Baseline	Day #1 Post-embolization	Day #2 Post-embolization	Day #3 Post-embolization
Troponin (*μ*g/mL)	0.0 ± 0.0	297.4 ± 58.0*	153.0 ± 38.8*	76.7 ± 19.8*

Troponin levels during baseline and daily post-embolization (mean ± standard deviation). Troponin levels peaked within twenty-four hours post-embolization and started decreasing towards baseline. (*n* = 5, *P < 0.05).
